# Predictors of prodromal Parkinson’s disease in young adult *Pink1−/−* rats

**DOI:** 10.3389/fnbeh.2022.867958

**Published:** 2022-09-12

**Authors:** Sarah A. Lechner, Jacob M. Welsch, Natalie K. Pahapill, Taylor A. R. Kaldenberg, Amy Regenbaum, Cynthia A. Kelm-Nelson

**Affiliations:** Department of Surgery, Division of Otolaryngology, University of Wisconsin-Madison, Madison, WI, United States

**Keywords:** Parkinson’s disease, rat, PINK1, ultrasonic vocalization (USV), anxiety, motor

## Abstract

Parkinson’s disease (PD) is a progressive, degenerative disease that affects nearly 10 million people worldwide. Hallmark limb motor signs and dopamine depletion have been well studied; however, few studies evaluating early stage, prodromal biology exist. *Pink1−/−* rats, a rodent model of PD mitochondrial dysfunction, exhibit early stage behavioral deficits, including vocal communication and anxiety, that progress during mid-to-late adulthood (6–12 months of age). Yet, the biological pathways and mechanisms that lead to prodromal dysfunction are not well understood. This study investigated the *Pink1−/−* rat in young adulthood (2 months of age). Mixed sex groups of *Pink1−/−* rats and wildtype (WT) controls were assayed for limb motor, anxiety, and vocal motor behaviors. A customized NanoString CodeSet, based on genetic work in later adulthood, was used to probe for the up regulation of genes involved in disease pathways and inflammation within the brainstem and vocal fold muscle. In summary, the data show sex- and genotype-differences in limb motor, anxiety, and vocal motor behaviors. Specifically, female *Pink1−/−* rats demonstrate less anxiety-like behavior compared to male *Pink1−/−* rats and female rats show increased locomotor activity compared to male rats. *Pink1−/−* rats also demonstrate prodromal ultrasonic vocalization dysfunction across all acoustic parameters and sex differences were present for intensity (loudness) and peak frequency. These data demonstrate a difference in phenotype in the *Pink1−/−* model. *Tuba1c* transcript level was identified as a key marker negatively correlated to ultrasonic vocalization at 2 months of age. Identifying genes, such as *Tuba1c*, may help determine early predictors of PD pathology in the *Pink1−/−* rat and serve as targets for future drug therapy studies.

## Introduction

Parkinson’s disease (PD) is a degenerative disorder of the central and peripheral nervous systems that impacts 1–3% of the world’s population (de Lau et al., 2004). The disease is characterized by multiple pathological hallmarks including misfolded α-synuclein protein, Lewy bodies, and neuroinflammation, as well as severe nigrostriatal dopamine loss in the mid-to-later stages of the disease (Chandra et al., 2006). Patients most often express clinical motor signs of PD, such as bradykinesia, resting tremors, rigidity, and gait imbalance which lead to a formal diagnosis. However, non-motor signs such as cranial sensorimotor impairments, olfactory dysfunctions, sleep disturbances, and anxiety manifest upwards of a decade before the emergence of limb motor signs and are hypothesized to be independent of dopamine loss (Ho et al., 1998; Pellicano et al., 2007; Poewe, 2008; Al-Qassabi et al., 2017; Fullard et al., 2017; Schapira et al., 2017). Although much is known regarding the neuropathology of the classical limb motor signs and dopamine loss, the biological pathways and mechanisms that lead to prodromal non-motor signs, specifically cranial sensorimotor dysfunction, are inadequately understood. Thus, studying early dysfunction in PD, outside of the classical dopamine framework, could identify new disease biomarkers leading to earlier diagnoses and the development of novel treatments.

A complete loss of function mutation to PTEN-induced putative kinase1 (*PINK1*) gene in humans induces the second most identified form of autosomal recessive, early onset PARK6 PD (Valente et al., 2004a,b). PINK1 participates in mitochondrial function, reactive oxygen radical scavenging, and mitophagy (Geisler et al., 2010). The loss of function mutation in *PINK1* leads to nigrostriatal dopaminergic cell death in the late stages, motor and non-motor deficits, and mitochondrial pathology (Li et al., 2005; Poole et al., 2008; Thomas and Cookson, 2009). In an analogous homozygous genetic rat knockout model (*Pink1−*/−), studies report early and progressive metabolic, mitochondrial, motor, and sensorimotor deficits and anxiety-like/affective behaviors (Dave et al., 2014; Grant et al., 2015; Cullen et al., 2018; Kelm-Nelson et al., 2018; Glass et al., 2019; Marquis et al., 2019; Stevenson et al., 2019; Hoffmeister et al., 2021a,b). Likewise, *Pink1−/−* rats also demonstrate neurobiological changes, such as increased oxidative stress detected via MRI imaging, within the olfactory system and hypothalamus at 3 months of age; consistent with human literature indicating olfaction and sleep dysfunction are present in the prodromal stage of PD (Ferris et al., 2018). Therefore, the *Pink1−*/− rat is a useful model for studying early behavioral differences between sexes, potential biomarkers, and gene expression differences in early stage PD (rat early adulthood).

The *Pink1−*/− rat behavioral phenotype has been well characterized over the last 10 years. *Pink1−*/− rats exhibit sex-specific limb motor deficits, including slowness of movement, that are present by 8 months of age (Grant et al., 2015; Marquis et al., 2019). Additionally, studies have shown that the *Pink1−*/− rat shows anxiety-like behavior between 8 and 12 months and early anhedonia at 2 months (Marquis et al., 2019; Hoffmeister et al., 2021b); these signs are analogous to early clinical manifestations in humans. Rat ultrasonic vocalizations (USVs) are used to model cranial sensorimotor (vocal motor) deficits in PD (Ciucci et al., 2007; Grant et al., 2015). Reduced intensity (loudness), a trademark voice deficit feature of PD pathology in humans, has been observed in both male and female *Pink1−*/− rats as early as 2 months of age (Grant et al., 2015; Marquis et al., 2019). However, later-stage differences in PD vocalization deficits are present between the *Pink1−/−* sexes. For example, at 8 months of age, male *Pink1−*/− rats show vocalization deficits including decreased intensity, bandwidth, and peak frequency (Grant et al., 2015), whereas female *Pink1−/−* rats do not display progressive degeneration in multiple acoustic variables such as bandwidth, call complexity, and call rate from 2 to 8 months (Marquis et al., 2019). To date, there has been no direct comparison between sexes within the same study and thus, studying sex as a biological variable is a critical aspect of the present work.

*Pink1−/−* rats also demonstrate physiological and molecular differences in the vocal fold muscle, thyroarytenoid (TA), which is responsible, in part, for vocal production. For instance, male *Pink1−*/− rats at 6 months of age demonstrate differences in myosin heavy chain composition and myofibril size in the TA muscle. *Pink1−/−* rats have increased numbers of centralized nuclei that are negatively correlated to vocalization loudness (unpublished data); this suggests a link between peripheral pathology and functional aspects of the rat vocalization (Glass et al., 2019). In addition, recent work shows that by 8 months of age, loss of *Pink1* influences gene pathways and neurochemistry within the brainstem (periaqueductal gray; vocal modulator) as well as within the TA muscle, including genes involved in Parkin-Ubiquitin proteasome degradation, MAPK signaling, and inflammatory pathways (Kelm-Nelson and Gammie, 2020; Lechner et al., 2021). However, it is not known whether the differential expression of these genes is present in early adulthood (i.e., 2 months) and has not yet been assessed in female *Pink1−/−* rats.

While previous bodies of work have assessed locomotor and vocal motor behavior in this model as longitudinal studies, this study is the first to explore the differences in locomotion, anxiety, and vocalization between *Pink1−/−* male and female rats as young adults (2 months of age). At present, no other study has investigated gene expression changes in the brainstem or vocal fold muscle (TA) at this early stage timepoint. Potential biomarkers and gene expression candidates have been previously identified in 8-month-old male rats and here, using a customized NanoString Code set, these gene transcripts were then probed at 2 months of age, and across sexes. We specifically hypothesized that genes involved in apoptosis, disease pathways (Parkinson’s, Alzheimer’s, Huntington’s, ALS), Parkin-Ubiquitin proteasome degradation, MAPK signaling, and inflammatory pathways would be differentially expressed in young adult *Pink1−/−* rats compared to WT controls, and sex-specific differences would be present, regardless of genotype. We hypothesized that these key genes would be identified as early predictors of PD pathology in the *Pink1−*/− rat.

## Materials and methods

### Animals and experimental design

A total of 24 Long Evans rats with a homozygous *Pink1−*/− knockout (*n* = 12 male, *n* = 12 female) and 24 wildtype (WT) control rats (*n* = 12 male, *n* = 12 female) (Envigo, Indianapolis, IN) were used in this study. A separate group of WT stimulus rats (*n* = 6 male, *n* = 6 female) (Charles River, Wilmington, MA) were used to elicit USVs, but were not included as study animals or part of the statistical analysis. All rats arrived at 4–6 weeks old and were pair-housed (same-sex, same-genotype) in standard polycarbonate cages (17 cm × 28 cm × 12 cm) with corncob bedding. Food and water were provided *ad libitum* throughout the study. Immediately upon arrival, all rats were immediately placed on a 12:12-h reverse light cycle. Rats were acclimated to study procedures and experimenter handling for 1 week prior to behavioral testing. All behavioral testing (see below) was performed under partial red-light illumination during the dark cycle to ensure they were in an alert state during testing.

All procedures and protocols (M006329-R01) were approved by the University of Wisconsin-Madison School of Medicine and Public Health Animal Care and Use Committee (IACUC) and were conducted in accordance with the NIH Guide for the Care and Use of Laboratory animals (National Institutes of Health, Bethesda, MA, United States).

### Body weights

Rats were weighed (g) once per week and prior to all behavioral assays using a calibrated digital scale.

### Female estrous staging

Female rat estrous stage has been shown to influence behavior and ultrasonic vocalization production (Matochik et al., 1992). Therefore, all behavioral testing and tissue collection occurred while the female rat was in the estrus stage of the estrous cycle. All female rats’ current stage of estrous cycle was determined daily by behavioral cues (ear wiggling, darting, lordosis) as well as cytodiagnosis following vaginal lavage. A pipette containing 0.20 mL of sterile saline was inserted approximately 5–10 mm into the vaginal orifice and flushed several times, then recollected by the pipette (Cora et al., 2015). The samples were mounted onto a slide, allowed to air dry, then stained with Wright’s Stain [Rapid Formula (Ricca, #9350)]. To determine stage of estrous cycle (4 stages: proestrus, metestrus, estrus, diestrus), cell density as well as the absence, presence, and proportion of cell types on each slide were analyzed by two raters using a confocal microscope (Olympus FV1000 Laser Scanning Confocal Microscope, Madison, Wisconsin). If a female was confirmed to be in the estrus phase, all behavioral testing and tissue collection was performed on the same day. Estrous swabbing was performed every day until each female rat was confirmed to be in the estrus stage and behavioral testing could be performed.

### Open field

Locomotion and anxiety-like behaviors (thigmotaxis) (Prut and Belzung, 2003; Seibenhener and Wooten, 2015) were assessed using an open field arena [60 cm × 60 cm surrounded by walls 40 cm in height (Maze Engineers, IL)]. Square grid crossings on the floor of the arena were used to track total distance traveled and time spent in the center vs. the periphery of the arena. Each rat was placed in the center of the arena and recorded over a 5 min interval with a Basler ac1300–06 (Basler GenIcam, Exton, PA) video-camera mounted above the arena. Number of entries into the center zone (#), time spent in the center zone (sec), and total distance traveled (cm) were analyzed using video-tracking software (Ethovision Version 4.0, Noldus Information Technology, Netherlands).

### Cylinder

A transparent cylinder (20 cm × 30 cm) was positioned on a piece of glass with a camera (Sony HDR-CX210) located below to assess spontaneous limb motor activity through the glass over a 1 min period (Fleming et al., 2004). Two raters, masked to genotype and sex, viewed recordings in slow motion to analyze the number of hindlimb and forelimb movements and number of rears and lands. Interrater reliability was over 0.95 for each measurement.

### Ultrasonic vocalization recording

Ultrasonic vocalization recording and analysis was performed identical to previous work (Grant et al., 2015, 2018; Kelm-Nelson et al., 2018; Marquis et al., 2019; Hoffmeister et al., 2021b). USVs were categorized [frequency modulated (FM) or simple] by independent raters masked to genotype and sex (see Ciucci et al., 2007, 2009; Johnson et al., 2011 for details). Total number of USVs and the percent of complex USVs (FM) were collected and analyzed. If rats produced fewer than 30 total USVs, they were removed from all statistical analyses. The average, maximum (max), and top 10 was calculated for all, simple, and FM ultrasonic vocalization duration (seconds-sec), bandwidth (hertz-Hz), intensity (loudness, decibel-dB), and peak frequency (hertz-Hz). In this study, the presented results (statistical analysis, results section, and corresponding graphs) are focused on FM call types. Analysis of all calls and simple calls showed similar statistical relationships and subsequently, the data are presented in Supplementary Tables 1–6.

### Euthanasia and tissue processing

Following behavioral testing at 2 months of age, all rats were deeply anesthetized with isoflurane and rapidly decapitated. Brains and whole larynges were grossly dissected and immediately frozen and stored at −80°C.

#### Brainstem dissection

Frozen brains were dissected as described in Dissection of Rodent Brain Regions (Spijker, 2011). Briefly, the frozen brain was bisected using a brain block (Kent Scientific) at approximately −7.5 Bregma. The cortex and cerebellum were removed with a forceps and surgical scissors and the spinal cord was trimmed as necessary (Heffner et al., 1980; Chiu et al., 2007). Dissected brainstem samples were kept frozen in microcentrifuge tubes at −80°C.

#### Thyroarytenoid dissection

Frozen larynges were viewed under a dissection microscope to visualize the thyroid cartilage, arytenoid cartilages, and epiglottis. Any extraneous tissues such as the epiglottis and adjacent mucosa were removed. The posterior larynx was bisected longitudinally through the thyroid cartilage to separate the left and right TAs at their insertion into the thyroid cartilage. Using a micro-forceps and micro-scissors, the TA muscles were isolated by removing them from all cartilaginous attachments (thyroid and arytenoid cartilages). Isolation of the TA muscle was performed as quickly as possible to avoid degradation of biological targets. Individual dissected samples from the left and right TA muscles were placed into separate Eppendorf microcentrifuge tubes and promptly frozen at −80°C until RNA processing.

#### RNA isolation

Sample order was randomized throughout the molecular portion of the study. An equal number of left or right TA samples were randomly selected for RNA extraction. TA and brainstem samples were homogenized with an electric sonic dismembrator (Thermo Fisher Scientific, Hampton, NH, United States) and the Bio-Rad Aurum Total RNA Fatty and Fibrous Tissue Kit (Catalog No. 732–6830; Bio-Rad, Hercules, CA, United States) was used for RNA extraction according to the manufacturer’s instructions. Total RNA was measured using a Nanodrop system (Thermo Fisher Scientific, Wilmington, DE, United States). All samples had an A260/A280 ratio that fell within the 1.8–2.20 range.

### NanoString and gene enrichment

The Custom CodeSet (Supplementary Table 7) was based on differential mRNA expression of genes from WT and *Pink1*−/− brainstem periaqueductal gray GSE150939 and vocal fold muscle GSE151209 datasets. The Custom NanoString CodeSet included 187 genes of interest, 5 housekeeping genes (*Actb*, *B2m*, *Gapdh*, *Pgk1*, *Ywaz*), and positive and negative controls from the manufacturer. To quantify expression of single transcripts with sensitive and reliable expression, samples were run using NanoString nCounter Technology (NanoString Technologies, Seattle, WA). Briefly, total RNA (100 ng) was used as input for nCounter sample preparation reactions and reactions were automated and performed as directed by the manufacturer. Each transcript was detected by a probe bound to tag-specific nCounter capture and barcoded reporter probes. Hybridized probes were purified and immobilized on a streptavidin-coated cartridge using the nCounter Prep Station. For each run, a high-density scan (600 fields of view) was performed. Counts below 20 were considered “not present.” Results were analyzed with the nCounter Digital Analyzer Software to count individual fluorescent barcodes and quantify target RNA molecules present in each sample. Raw NanoString counts were background adjusted with a Poisson correction based on the negative control spikes included in each run. This was followed by a technical normalization using the 5 housekeeping genes included in each run. Fold-change expression and *p*-values were calculated by linear regression analysis using negative binomial or log-linear models with WT as the baseline value. *P*-values were corrected for multiple comparisons using the Benjamini-Yekutieli method. The average number of transcripts of WT (*n* = 3/sex) and *Pink1*−/− (*n* = 3/sex) experimental replicates was calculated. Transcript expression for individual rats (*n* = 6) was used to correlate to behavior. Gene ontology and pathway enrichment analysis was performed using ENRICHR and the KEGG 2021 Human and Disease Perturbations from GEO modules. Additionally, the top up- and down-regulated gene lists were used in Drug Perturbations from GEO Up and Down, respectively, to identify drug repurposing compounds for future work.

#### Tuba1c RT qPCR and western blot

To verify the top candidate, *Tuba1c*, from the NanoString study, RNA from each sample was converted into single-stranded cDNA using the Invitrogen SuperScript III kit (First Strand, Invitrogen, 18080, Carlsbad, CA, United States). NCBI Primer Blast was used to design custom primers from Integrated DNA Technologies (25 nmole DNA oligo with standard desalting) for *Gapdh* (Forward: GGATACTGAGAGCAAGAGAGA, Reverse: TTATGGGGTCTGGGATGGAA) and *Tuba1c* (Forward: AGGAGACGATGAGGGTGAAG, Reverse: ACGCAAGGACAAAGATGAGAC) (IDT, Coralville, Iowa, United States). Netprimer (PREMIER Biosoft, Palo Alto, CA, United States) was used to examine secondary structure of all primers to dimers and non-specific amplification products. Specificity for each primer pair was confirmed using melt curve analysis with a primer runs which yielded single peak melt curves. Relative gene expression was determined using real-time (RT) quantitative PCR (RT-qPCR) analysis following the MIQE guidelines for PCR experiments. All samples were run on one plate and were prepared in reaction tubes containing the respective sample cDNA, nuclease-free water, characterized forward and reverse primers (5 μM concentration) and SsoFast EvaGreen Supermix (Catalog No. 172-5201). Five standards were run (1:10 serial dilutions, starting at 500 ng/μL) with a non-template negative control for both genes, respectively. Samples were run in triplicate. The plate was run with Bio-Rad programming, read, and analyzed with the Bio-Rad CFX96 Touch Real-Time PCR Detection System (Catalog No. 185-5195, Bio-Rad, Hercules, CA, United States), and the relative quantity (delta Cq) of *Tuba1c* was determined using the Bio-Rad software for each sex/genotype.

Additionally, hemi-brainstem tissue samples for protein analysis (WT males = 5, *Pink1*−/− males = 5; WT females = 4, *Pink1*−/− females = 5) were homogenized using 1 mL of lysis buffer [N-PER Neuronal Protein Extraction Buffer (Thermo Fisher Scientific, Rockford, IL, United States) including a cocktail of protease (Sigma Aldrich, St. Louis, MO, United States), phosphatase inhibitors (Sigma Aldrich, St. Louis, MO, United States) and 200 mM PMSF (Sigma Aldrich, St. Louis, MO, United States)]. Samples were incubated on ice for 60 min and centrifuged at 12,000 rpm for 10 min at 4°C. Supernatant was collected, and total protein concentrations were quantitatively determined using a bicinchoninic acid protein assay (BCA Protein Assay Kit; Thermo Fisher Scientific Pierce, Rockford, IL, United States) using the manufacturer’s instructions. Supernatant was mixed with a pre-calculated volume of 2 × Laemmli buffer (Bio-Rad, #161–0737) with 2-mercaptoethanol. Extracted protein samples (50 μg of total protein from each rat as determined by BCA assay analysis) were denatured at 95°C for 5 min, and lysates were resolved on a Criterion Precast Gel (4–20% gradient Tris– HCl-polyacrylamide gels, 1.0 mm, 12 × 2 Well Comb, Bio-Rad, #3450032). Male and female samples were run on separate blots. Prestained protein standards (Precision Plus Protein Dual Xtra Standards, Bio-Rad, #161–0377) were included on gels as molecular mass markers. Mouse lysate (20 μg) was run as a control (Cell Signaling, 55330S) on each blot. Samples were subjected to electrophoresis in 10 × Tris-buffered saline buffer with glycine (TBS, Bio-Rad, #161–0771) for 1:15 h at 125 V and then transferred in 10 × TBS with glycine (Bio-Rad, #170–6435) with 20% methanol for 2 h at 80 V onto Immobilon-P transfer Membrane (0.45 μm pore size; Millipore, IPVH00010). Membranes were blocked with filtered 5% Bovine Serum Albumin (BSA, Thermo Fisher Scientific, #BP-1600) in Tris buffered saline containing 0.1% Tween-20 (TBS-T) for 1 h. Blots were probed with primary antibodies (rabbit anti-Tuba1c, 1:2,500, Abcam ab222849) and loading control (mouse anti-GAPDH, 1:25K, Proteintech #60004) in TBST containing filtered 1% BSA overnight (minimum 16 h) at 4°C with constant agitation. Following primary antibody incubation, blots were washed in TBS-T 6 × 10 min and then probed with horseradish peroxidase-conjugated anti-rabbit IgG (1:5,000 dilution, Cell Signaling Technology Inc., #7074S) and anti-mouse IgG (1:10,000 dilution, Cell Signaling Technology Inc., #7076S). Blots were washed in TBS-T 6 × 10 min and enhanced chemiluminescence substrate with Super Signal West Pico (5 min, Thermo Fisher Scientific, #34080) was used to develop immunoblots using a ChemiDoc-IT2 Imager (UVP, LLC). ImageJ (National Institutes of Health) was used to analyze grayscale band density normalized to GAPDH internal controls on each blot.

### Statistical analysis

Statistical analyses were conducted using SigmaPlot^®^ 13.0 (Systat Software, Inc., San Jose, CA). Unless otherwise indicated, a two-way Analysis of Variance (ANOVA) was used to make comparisons for behavioral testing dependent variables (described above) with independent variables being genotype (WT, *Pink1−/−*) and sex (male, female). Variables were transformed (either rank or square root transformed) if data failed to adhere to normality (Shapiro-Wilk test) and equal variance (Levene’s test) assumptions for ANOVA. Main effects and interactions were examined. *Post hoc* analysis was performed with Fisher’s Least Significant Difference (LSD). Statistical analysis for NanoString is described above. ANOVA was used to analyzed sex/genotype RT-qPCR data, and Mann-Whitney U were used to analyze western blot data between sexes. The critical level for significance was set *a priori* at 0.05. Pearson correlation analysis was performed for all behavioral variables and transcript expression.

## Results

Means and standard error of the means (SEM) for behavioral data are presented in Table 1.

**TABLE 1:** Behavioral means (SEM).

Behavioral variable	Units	Male		Female	
		WT	*Pink1−/−*	WT	*Pink1−/−*
Bodyweight	G	229.83 (2.33)	254.50 (4.07)	205.42 (4.36)	188.33 (6.78)
Cylinder hindlimb	#	8.75 (1.19)	9.00 (0.71)	15.67 (1.72)	15.75 (1.47)
Cylinder forelimb	#	14.75 (1.04)	15.75 (0.61)	21.50 (2.83)	28.50 (2.52)
Rears and lands	#	17.67 (1.56)	16.67 (0.74)	20.58 (1.63)	19.17 (0.90)
USV total calls	#	141.00 (14.90)	108.70 (7.94)	77.90 (9.20)	95.10 (17.33)
USV % complex	%	0.72 (0.04)	0.82 (0.02)	0.66 (0.06)	0.80 (0.03)
OF time in center zone	Sec	44.78 (7.91)	36.94 (5.47)	36.74 (2.53)	79.57 (9.83)
OF number of entries into center zone	#	17.75 (2.76)	18.25 (1.74)	20.67 (2.21)	29.33 (2.04)
OF total movement	Cm	2798.63 (238.03)	3119.66 (164.96)	3024.88 (237.85)	3635.58 (170.03)

Mean [standard error of the mean (SEM)] for behavioral variables for each genotype and sex. G, grams; USV, ultrasonic vocalization; OF, open field; sec, second; cm, centimeter.

### Body weight

**FIGURE 1:**
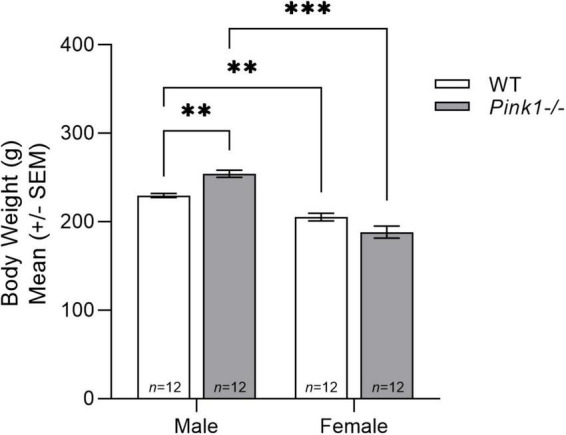
Body weight. Means (± standard error of the mean, SEM) of male and female rat body weight (grams, g) in wildtype (WT, white bar) compared to *Pink1–/–* (light gray bar) at 2 months of age. Bars indicate statistical significance between groups (sex and genotype) with asterisks showing levels of significance (^∗∗^*p* < 0.01, ^∗∗∗^*p* < 0.001).

There was a significant genotype (WT, *Pink1−*/−) by sex (male, female) interaction for body weight [*F*(1, 44) = 20.049, *p* < 0.001]. Specifically, WT males were significantly heavier than WT females (*p* < 0.001). Additionally, *Pink1−*/− males were heavier than *Pink1−*/− females (*p* < 0.001). Within each sex, *Pink1−*/− males were heavier compared to WT males (*p* < 0.001). However, WT females were heavier than *Pink1−*/− females (*p* = 0.013) (Figure 1).

### Open field

**FIGURE 2:**
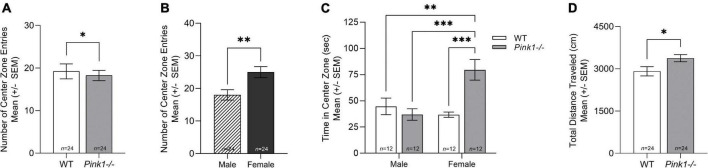
Open field behavior. **(A)** Means (± standard error of the mean, SEM) of wildtype (WT, white bar) compared to *Pink1–/–* (light gray bar) for number of entries into the center zone of the open field apparatus. **(B)** Average number of center zone entries of male (lined bar) compared to female (dark gray bar). **(C)** Average time spent in the center zone (± SEM) of all rats. **(D)** Average total distance traveled in the open field of WT compared to *Pink1–/–*. Bars indicate statistical significance between groups with asterisks showing levels of significance (^∗^*p* < 0.05; ^∗∗∗^*p* < 0.001).

There was no significant interaction of genotype and sex for the number of entries into the center zone [*F*(1, 44) = 3.386, *p* = 0.072]. There was a main effect of genotype [*F*(1, 44) = 4.266, *p* = 0.045]; *Pink1−*/− rats entered the center zone fewer times than WT controls (Figure 2A). Additionally, there was a main effect of sex [*F*(1, 44) = 9.951, *p* = 0.003] where female rats had significantly more entries into the center zone compared to males (Figure 2B). There was a significant interaction of genotype and sex for the time spent in the center zone [*F*(1, 44) = 11.619, *p* = 0.001]. *Post hoc* analysis demonstrated that *Pink1−*/− females had increased time in the center zone compared to *Pink1−*/− males (*p* < 0.0001), WT males (*p* = 0.001), and WT females (*p* < 0.0001) (Figure 2C). There was no significant interaction of genotype and sex for the total distance traveled in the open field [*F*(1, 44) = 1.435, *p* = 0.237]. There was a main effect of genotype [*F*(1, 44) = 6.523, *p* = 0.014]; *Pink1−*/− rats had increased number of movements compared to WT controls (Figure 2D). There was no significant main effect of sex (*p* > 0.05).

### Cylinder

**FIGURE 3:**
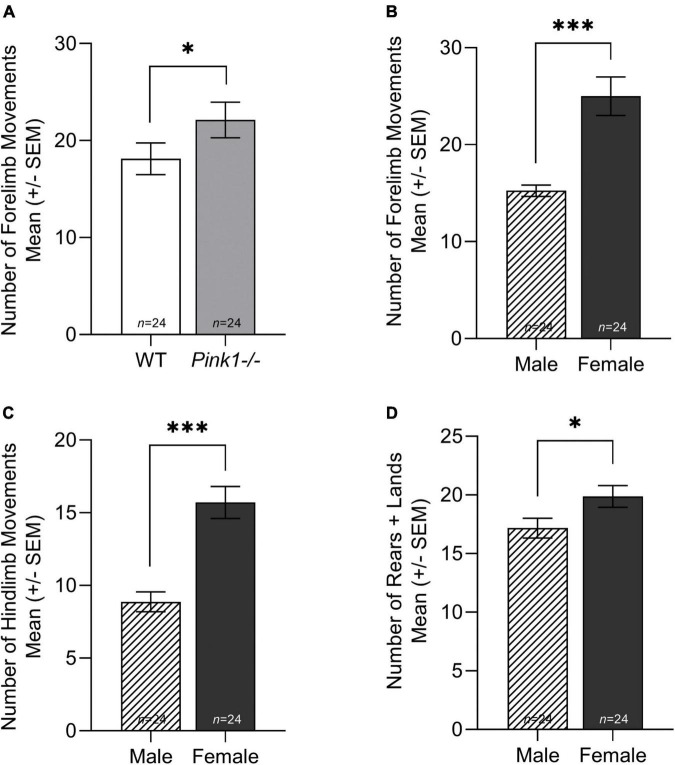
Cylinder movement. **(A)** Average number of forelimb movements of wildtype (WT, white bar) and *Pink1–/–* (light gray bar) (± standard error of the mean, SEM). **(B)** Average number of forelimb movements of male (lined bar) and female (dark gray bar) rats (± SEM). **(C)** Average number of hindlimb movements of male and female rats (± SEM). **(D)** Average total number of rears + lands by male and female rats (± SEM). Bars indicate statistical significance between groups with asterisks showing levels of significance (^∗^*p* < 0.05; ^∗∗^*p* < 0.01, ^∗∗∗^*p* < 0.001).

There was no significant interaction between genotype and sex for number of forelimb movements [*F*(1, 44) = 2.173, *p* = 0.148], hindlimb movements [*F*(1, 44) = 0.00394, *p* = 0.950], or rears and lands [*F*(1, 44) = 0.0296, *p* = 0.864]. There was a main effect of genotype for number of forelimb movements [*F*(1, 44) = 4.50, *p* = 0.038]; overall, *Pink1−/−* rats had more forelimb movements than WT rats (Figure 3A). There was no main effect of genotype for number of hindlimb movements or rears and lands (*p* > 0.05 for both). For all cylinder variables, there was a main effect of sex (*p* < 0.05). Female rats had significantly more forelimb movements [*F*(1, 44) = 32.850, *p* < 0.001] (Figure 3B), hindlimb movements [*F*(1, 44) = 26.477, *p* < 0.001] (Figure 3C), and rears and lands [*F*(1, 44) = 5.069, *p* = 0.029] compared to males (Figure 3D).

### Ultrasonic vocalization

Means and SEM for FM ultrasonic vocalization non-acoustic parameters are also presented in Table 1. Means and SEM for average, maximum (max), and top 10 values for FM acoustic parameters are presented in Table 2. Interaction effect and main effect F and *P*-values for FM acoustic parameters are presented in Supplementary Tables 8, 9.

**TABLE 2:** Frequency modulated (FM) calls—Means (SEM).

	Acoustic parameter/unit	Male		Female	
		WT	*Pink1−/−*	WT	*Pink1−/−*
Average	Duration (sec)	0.042 (0.002)	0.031 (0.002)	0.042 (0.005)	0.032 (0.002)
	Bandwidth (Hz)	22023.01 (2078.07)	14222.81 (1266.48)	21720.04 (1188.19)	17453.45 (1720.07)
	Intensity (dB)	−47.68 (0.81)	−44.52 (0.56)	−49.83 (1.27)	−47.44 (0.59)
	Peak frequency (Hz)	56006.79 (777.96)	48262.40 (1318.54)	63758.55 (1415.30)	56291.80 (825.70)
Maximum	Duration	0.120 (0.016)	0.112 (0.043)	0.089 (0.025)	0.087 (0.015)
	Bandwidth	50258.33 (3973.08)	35200.00 (3904.16)	39290.00 (3056.16)	33040.00 (2889.64)
	Intensity	−33.67 (0.72)	−31.98 (0.72)	−36.80 (2.23)	−35.60 (1.12)
	Peak frequency	70958.33 (1717.84)	59630.00 (2482.52)	75490.00 (1345.48)	68070.00 (2279.53)
Top 10	Duration	0.071 (0.007)	0.048 (0.008)	0.057 (0.011)	0.048 (0.006)
	Bandwidth	36416.67 (3244.35)	24258.00 (2535.32)	28014.10 (1938.52)	24693.00 (3260.96)
	Intensity	−38.05 (1.37)	−36.43 (0.63)	−44.25 (2.16)	−41.60 (1.42)
	Peak frequency	65051.67 (1443.29)	52417.00 (1923.91)	69200.29 (1175.30)	61741.00 (1994.99)

Mean [standard error of the mean (SEM)] for acoustic parameters of frequency modulated (FM) ultrasonic vocalizations for each genotype and sex. Sec, second; Hz, Hertz; dB, decibel.

#### Total number of calls

**FIGURE 4:**
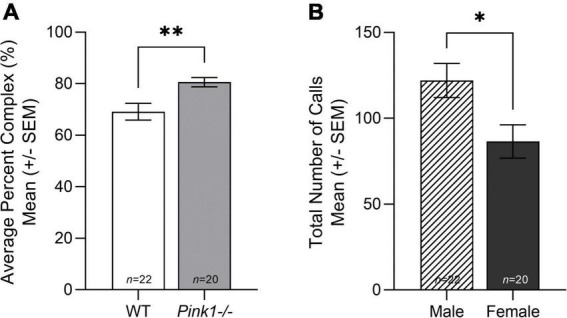
Total number and complexity of all ultrasonic vocalizations. **(A)** Average total number of calls (± standard error of the mean, SEM) made by male (lined bar) compared to female (dark gray bar) rats. **(B)** Average percent complex of wildtype (WT, white bar) and *Pink1–/–* (light gray bar) rats. Bars indicate statistical significance between groups with asterisks showing levels of significance (^∗^*p* < 0.05; ^∗∗^*p* < 0.01).

There was no significant interaction between genotype and sex for total number of calls [*F*(1, 38) = 2.287, *p* = 0.139]. There was no significant main effect of genotype (*p* > 0.05), but there was a main effect of sex [*F*(1, 38) = 7.446, *p* = 0.010]. Male rats produced significantly more calls than female rats regardless of genotype (Figure 4A).

#### Percent complex

There was no significant interaction between genotype and sex for percent complex calls [*F*(1, 38) = 0.257, *p* = 0.615], but there was a significant main effect of genotype [*F*(1, 38) = 9.135, *p* = 0.004]. *Pink1−/−* rats had a higher percentage of complex calls than WT rats (Figure 4B).

#### Duration

**FIGURE 5:**
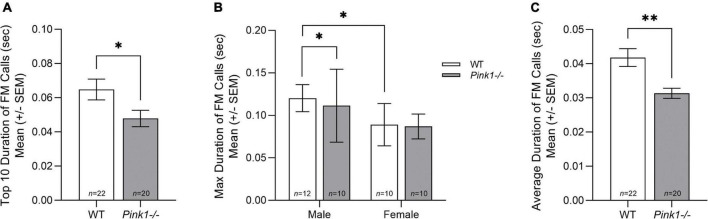
Duration of frequency modulated ultrasonic vocalizations. Means (± standard error of the mean, SEM) of **(A)** average, **(B)** maximum (max), and **(C)** top 10 duration (sec) of all frequency modulated (FM) calls produced by wildtype (WT, white bar) and *Pink1–/–* (light gray bar) rats. Bars indicate statistical significance between groups with asterisks showing levels of significance (^∗^*p* < 0.05; ^∗∗^*p* < 0.01).

There was no significant interaction between genotype and sex for average duration [*F*(1, 38) = 2.207, *p* = 0.146] and top 10 duration [*F*(1, 38) = 2.060, *p* = 0.159] of FM calls. However, there was a significant interaction between genotype and sex for max duration [*F*(1, 38) = 4.908, *p* = 0.033] of FM calls. Specifically, male WT rats have significantly longer max duration than male *Pink1−/−* rats, but there is no difference in max duration of FM calls in female WT rats and *Pink1−/−* rats (Figure 5A). Male WT rats also had significantly longer max duration of FM calls than female WT rats. For average duration [*F*(1, 38) = 15.800, *p* < 0.001] and top 10 duration [*F*(1, 38) = 4.967, *p* = 0.032], there was a significant main effect of genotype. WT rats had significantly longer FM calls than *Pink1−/−* rats (Figures 5B,C). There was no significant main effect of sex for average, max, and top 10 duration (*p* > 0.05).

#### Bandwidth

**FIGURE 6:**
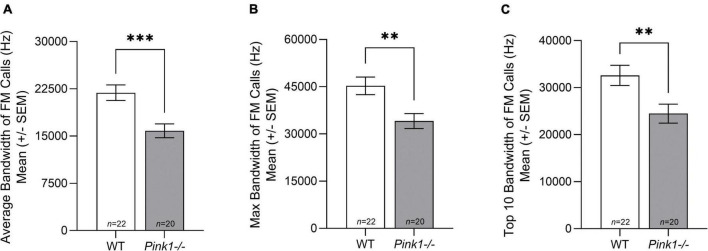
Bandwidth of frequency modulated ultrasonic vocalizations. Means (± standard error of the mean, SEM) of **(A)** average, **(B)** maximum (max), and **(C)** top 10 bandwidth (hertz, Hz) of all frequency modulated (FM) calls produced by wildtype (WT, white bar) and *Pink1–/–* (light gray bar) rats. Bars indicate statistical significance between groups with asterisks showing levels of significance (^∗∗^*p* < 0.01, ^∗∗∗^*p* < 0.001).

There was no significant interaction between genotype and sex for bandwidth of FM calls; average bandwidth [*F*(1, 38) = 1.117, *p* = 0.297], max bandwidth [*F*(1, 38) = 1.524, *p* = 0.225], top 10 bandwidth [*F*(1, 38) = 2.373, *p* = 0.132]. However, for average [*F*(1, 38) = 13.031, *p* < 0.001], max [*F*(1, 38) = 8.917, *p* = 0.005], and top 10 [*F*(1, 38) = 7.279, *p* = 0.010] bandwidth, there was a significant main effect of genotype. WT rats had significantly greater bandwidth FM calls than *Pink1−/−* rats (Figure 6). There was no significant main effect of sex for average, max, and top 10 bandwidth (*p* > 0.05).

#### Intensity

**FIGURE 7:**
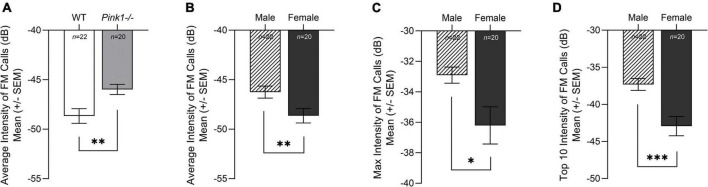
Intensity of frequency modulated ultrasonic vocalizations. **(A)** Means (± standard error of the mean, SEM) of average intensity (decibels, dB, negative scale) of all frequency modulated (FM) calls produced by wildtype (WT, white bar) compared to *Pink1-/-* rats (light gray bar). *Pink1-/-* rats produced calls with greater intensity (less negative, louder) than WT rats. Means (± SEM) of average **(B)**, maximum (max) **(C)**, and top 10 **(D)** intensity of all FM calls produced by male (lined bar) and female (dark gray bar) rats. Bars indicate statistical significance between groups with asterisks showing levels of significance (^∗^*p* < 0.05; ^∗∗^*p* < 0.01, ^∗∗∗^*p* < 0.001).

There was no significant interaction between genotype and sex for intensity of FM calls; average intensity [*F*(1, 38) = 0.201, *p* = 0.657], max intensity [*F*(1, 38) = 0.980, *p* = 0.328], top 10 intensity [*F*(1, 38) = 0.119, *p* = 0.732]. For average intensity [*F*(1, 38) = 10.504, *p* = 0.002], there was a main effect of genotype. *Pink1−/−* rats had greater intensity (loudness) of FM calls than WT rats (Figure 7A). However, for average [*F*(1, 38) = 8.760, *p* = 0.005], max [*F*(1, 38) = 5.915, *p* = 0.020], and top 10 [*F*(1, 38) = 14.523, *p* < 0.001] intensity, there was a significant main effect of sex. Male rats had significantly greater intensity of FM calls than female rats regardless of genotype (Figure 7).

#### Peak frequency

**FIGURE 8:**
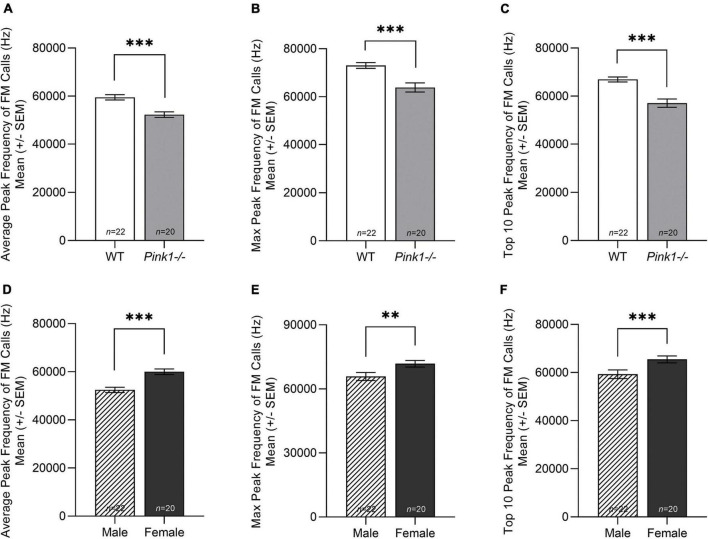
Peak frequency of frequency modulated ultrasonic vocalizations. Means (± standard error of the mean, SEM) of **(A)** average, **(B)** maximum (max), and **(C)** top 10 peak frequency (hertz, Hz) of all frequency modulated (FM) calls produced by wildtype (WT, white bar) compared to *Pink1-/-* (light gray bar) rats. Means (± SEM) of **(D)** average, **(E)** maximum (max), and **(F)** top 10 peak frequency of all FM calls produced by male (lined bar) and female (dark gray bar) rats. Bars indicate statistical significance between groups with asterisks showing levels of significance (^∗∗^*p* < 0.01, ^∗∗∗^*p* < 0.001).

There was no significant interaction between genotype and sex for peak frequency of FM calls; average peak frequency [*F*(1, 38) = 0.016, *p* = 0.900], max peak frequency [*F*(1, 38) = 0.962, *p* = 0.333], top 10 peak frequency [*F*(1, 38) = 2.435, *p* = 0.127]. However, for all three parameters, there was a significant main effect of genotype and sex. WT rats had significantly greater peak frequency of FM calls than *Pink1−/−* rats; average peak frequency [*F*(1, 38) = 47.985, *p* < 0.001] (Figure 8A), max peak frequency [*F*(1, 38) = 22.126, *p* < 0.001] (Figure 8B), and top 10 peak frequency [*F*(1, 38) = 36.700, *p* < 0.001] (Figure 8C). Female rats had significantly greater peak frequency of FM calls than male rats; average peak frequency [*F*(1, 38) = 51.649, *p* < 0.001] (Figure 8D), max peak frequency [*F*(1, 38) = 10.592, *p* = 0.002] (Figure 8E), and top 10 peak frequency [*F*(1, 38) = 16.498, *p* < 0.001] regardless of genotype (Figure 8F).

#### Range of intensity

There was no significant interaction between genotype and sex for range of intensity [*F*(1, 38) = 0.00253, *p* = 0.960] of FM calls. There was also no main effect of genotype [*F*(1, 38) = 0.658, *p* = 0.422] or sex [*F*(1, 38) = 1.928, *p* = 0.173].

### NanoString

#### Brainstem transcript expression

Each transcript table provides the gene name, accession number, direction of expression, fold change (FDR), *p*-value, and t statistics. Gene enrichment and drug repurposing tables include *p*-value, scores, and gene lists. Gene lists and enrichment tables are sorted by *p-*value. Due to small sample sizes used in NanoString, fold change is often below the standard 2.0 cutoff.

**FIGURE 9:**
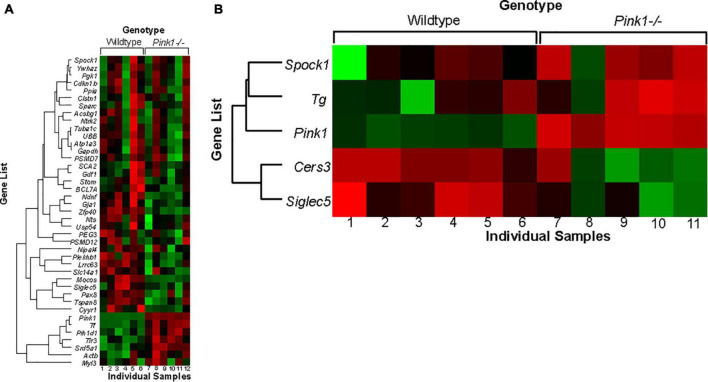
Heat map of significant genes found in the brainstem. **(A)** Brainstem **(B)** TA muscle heat maps of significant genes. The NanoString nSolver Analysis Software was used to generate an agglomerative cluster heat map using hierarchical cluster analysis with Pearson correlations on the log count values to measure distance between genes using their average linkage. The red shading indicates low gene expression relative to the average expression, black shading indicates average expression, and green shading indicates high gene expression. All WT samples were clustered on the left and all *Pink1–*/– samples were clustered on the right of the panel.

**FIGURE 10:**
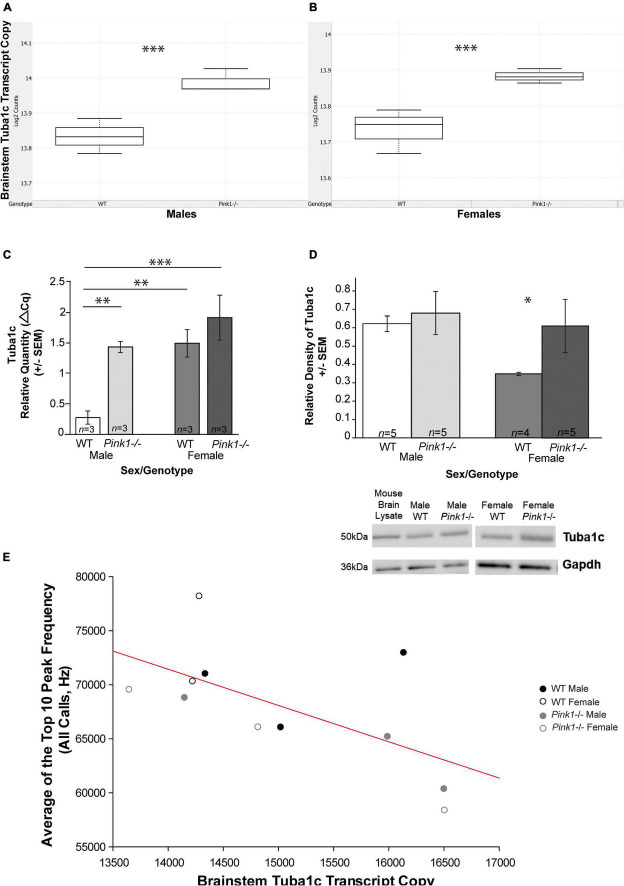
Brainstem *Tuba1c* mRNA transcript copy and vocalization peak frequency correlations. NanoString nCounter *Tuba1c* gene expression (Log2 Counts, *y*-axis) is upregulated in *Pink1*-/- rats compared to wildtype (WT) controls (*x*-axis) in both **(A)** males (***p < 0.001; *n* = 3 WT; *n* = 3 *Pink1*-/-) and **(B)** females (*** *p* < 0.001; *n* = 2 WT, *n* = 3 *Pink1*-/-). For box plots, the boundary of the box closest to zero indicates the 25th percentile. The line within the box marks the median and the boundary of the box farthest from zero indicates the 75th percentile. Whiskers (error bars) above and below the box indicate the 90th and 10th percentiles. **(C)** Tuba1c relative mRNA quantity (RT qPCR) in the brainstem of male and female rats (WT and *Pink1*-/-). Asterisk demonstrates significant differences (**p < 0.01; ***p < 0.001). Error bars are SEM, sample sizes are noted in each bar. **(D)** Tuba1c relative protein density from western blot analysis. Net protein concentrations were normalized to loading control (Gapdh) for male and female rats (WT and *Pink1*-/-). Error bars are standard error of the mean, sample sizes are noted in each bar. Asterisk demonstrates significant differences (*p < 0.05). Representative western blot bands for all groups for Tuba1c and reference control Gapdh. Molecular weight (kDa) and mouse brain lysate shown at left for comparison. **(E)** The average of the top 10 peak frequency of all ultrasonic vocalizations (hertz, Hz, *y*-axis) is negatively correlated to brainstem *Tuba1c* transcript copy (Log2 counts, *x*-axis) (*n* = 11). Colored dots indicate sex (males = black; females = gray) and genotype distinctions (closed circles = male; open circles = female). Red regression line is significant correlation (*p* < 0.05).

*Pink1* was the most down-regulated gene in the brainstem in *Pink1−*/− rats compared to WT controls (FDR = −22.2; *p* < 0.0001); there were more up-regulated genes (35) compared to down-regulated (6) in this dataset (Table 3 and Figure 9A). Using the ENRICHR software on the list of differentially expressed genes, there were several significant biological pathways (KEGG 2021 Human gene enrichment) that included multiple degenerative disease pathways including Alzheimer’s, PD, and Huntington’s as well as multiple signaling pathways including proteasome, GMP-PKG, cytokine, HIF, and MAPK (Table 4). This was similar to the overall CodeSet enrichment analysis (Supplementary Table 10). Using the list of significantly up-regulated genes, a list of drug compounds that target the reverse/down-regulation (Drug Perturbations GEO DOWN) are listed in Supplementary Table 11; of interest these include candesartan, phenytoin, and resveratrol. In both the gene list, enrichment, and drug repurposing lists *Tuba1c* (Tubulin Alpha 1c; Figures 10A,B) was a top gene candidate. RT-qPCR confirmed NanoString findings (Figure 10C; *F*(3, 8) = 10.07, *p* = 0.004). Specifically, there was increased gene expression of *Tuba1c* in *Pink1−*/− males compared to WT males (*p* < 0.01), and in females of both genotypes compared to WT males (*p* < 0.01). Female *Pink1−*/− also had increased Tuba1c relative protein (*T* = 10, *p* = 0.016; Figure 10D).

**TABLE 3:** Brainstem gene expression differences by genotype.

Gene	Accession #	Direction	*Pink1*−/− vs. WT FDR	*p*-value of: *Pink1*−/− vs. WT	t-statistic of: *Pink1*−/− vs. WT
Pink1	NM_001106694.1	Down	−22.22	0.00000001	−48.905159
Mocos	NM_001108425.1	Up	1.81	0.0000011	10.84265518
Zfp40	NM_001168642.1	Up	1.77	0.00000307	11.00094986
UBB	NM_138895.1	Up	1.1	0.00001562	9.31716347
Atp1a3	NM_012506.1	Up	1.13	0.00011065	6.5275321
Srd5a1	NM_017070.3	Down	−1.63	0.00012045	−6.22791624
Ndnf	XM_008763014.2	Up	1.36	0.00017583	6.38862944
Tuba1c	NM_001011995.1	Up	1.11	0.00027619	6.14361382
Gja1	NM_012567.2	Up	1.16	0.00065674	4.86529875
Tf	NM_001013110.1	Down	−1.35	0.00076893	−6.36818075
Lrrc63	NM_001024803.1	Up	1.38	0.00296313	4.58920145
PSMD7	NM_001107426.1	Up	1.08	0.00408769	3.70338106
Gapdh	NM_017008.2	Up	1.09	0.00473248	4.03119087
Nts	NM_001102381.1	Up	1.24	0.00656612	3.61494422
Mylk3	NM_001110810.1	Up	1.46	0.00719919	3.72874498
Ywhaz	NM_013011.3	Up	1.06	0.00734561	3.37146139
Pih1d1	NM_001024868.1	Down	−1.31	0.00759081	−3.51087379
Tlr3	NM_198791.1	Down	−1.29	0.00931468	−3.27550268
Slc14a1	NM_019346.2	Up	1.15	0.0113963	3.09560657
Acsbg1	NM_134389.1	Up	1.08	0.01210551	3.23836994
Tspan8	NM_133526.1	Up	1.34	0.01379933	3.01218724
BCL7A	XM_017598515.1	Up	1.18	0.014358	3.07249665
Ppia	NM_017101.1	Up	1.06	0.01437243	3.03436589
Actb	NM_031144.2	Down	−1.15	0.01516198	−3.06517577
Spock1	NM_001271297.1	Up	1.06	0.01686467	2.98241401
Stom	NM_001011965.1	Up	1.25	0.01706577	2.97140431
Pax8	NM_031141.2	Up	1.22	0.01898118	3.03792095
Plekhb1	NM_172033.2	Up	1.19	0.01921703	3.22909093
Usp54	NM_001008863.3	Up	1.1	0.02136607	2.7656312
Cyyr1	NM_001013980.1	Up	1.33	0.02297239	3.12222385
Cdkn1b	NM_031762.3	Up	1.08	0.02387715	2.9503665
Clstn1	NM_001007092.1	Up	1.07	0.0263533	2.65329814
Sparc	NM_012656.1	Up	1.08	0.02672601	2.59813666
PEG3	NM_001304816.1	Up	1.1	0.0419631	2.54467154
Nipal4	NM_001106995.1	Up	1.21	0.04246664	2.37983584
Pgk1	NM_053291.3	Up	1.04	0.04251458	2.34779787
Ntrk2	NM_012731.1	Up	1.07	0.04426007	2.4386735
PSMD12	NM_001005875.1	Up	1.08	0.05215243	2.22306418
SCA2	XM_003752609.1	Up	1.08	0.05275894	2.24228358
Gdf1	NM_001044240.2	Up	1.12	0.05407765	2.19625545
Siglec5	NM_001106249.2	Up	1.23	0.05423017	2.34178782

**TABLE 4:** KEGG 2021 human gene enrichment, upregulated genes in brainstem by genotype.

KEGG 2021 human UP genes	*p*-value	Combined Score	Genes
Alzheimer’s disease	4.63E-04	51.76815498	TUBA1C; PSMD12; PSMD7; IRS2; GAPDH; SNCA
Parkinson’s disease	5.53E-04	61.8028752	TUBA1C; PSMD12; PSMD7; UBB; SNCA
Transcriptional misregulation in cancer	0.00179	53.18437419	CDKN1B; PAX8; PAX5; RUNX2
Thyroid cancer	0.004485	118.3044422	TCF7L2; PAX8
Proteasome	0.006863	86.66326217	PSMD12; PSMD7
Pathways of neurodegeneration	0.008999	19.91848207	TUBA1C; PSMD12; PSMD7; UBB; SNCA
cGMP-PKG signaling pathway	0.010386	32.40671162	ATP1A3; IRS2; MYLK3
Alcoholism	0.013871	27.17618411	NTRK2; DDC; SLC29A2
Adipocytokine signaling pathway	0.014955	47.95995568	IRS2; ACSBG1
Epstein-Barr virus infection	0.017263	23.69391643	PSMD12; CDKN1B; PSMD7
Thyroid hormone synthesis	0.017508	42.35395328	PAX8; ATP1A3
Gastric acid secretion	0.017951	41.52173636	ATP1A3; MYLK3
Arrhythmogenic right ventricular cardiomyopathy	0.018398	40.71531034	TCF7L2; GJA1
Gap junction	0.023621	33.26826037	TUBA1C; GJA1
Prostate cancer	0.028293	28.65255993	TCF7L2; CDKN1B
Salmonella infection	0.029693	16.56670562	TUBA1C; TCF7L2; GAPDH
HIF-1 signaling pathway	0.035044	23.89781872	CDKN1B; GAPDH
Serotonergic synapse	0.03742	22.58108156	DDC; SLC6A4
Prion disease	0.037459	14.07978307	TUBA1C; PSMD12; PSMD7
Cell cycle	0.044261	19.48432548	CDKN1B; YWHAZ
Phenylalanine metabolism	0.044939	72.91477862	DDC
MAPK signaling pathway	0.045027	12.31864156	NTRK2; PPM1B; HSPB1
Fatty acid biosynthesis	0.04752	67.38709029	ACSBG1
FoxO signaling pathway	0.048838	17.83911121	CDKN1B; IRS2
Huntington disease	0.049669	11.44937317	TUBA1C; PSMD12; PSMD7

There were few sex differences within the brainstem of WT and *Pink1−*/− animals. For example, male *Pink1−*/− rats had 21 differentially expressed genes and female *Pink1−*/− rats had 17. In both sexes, there were more up-regulated genes than down-regulated; several genes overlapped including *Ubb*, *Tuba1c*, *Ndnf*, *Srd5a1*, *Zfp40*, *Mocos*, and *Atp1a3.* Using the KEGG 2021 Human ENRICHR database, the top pathways in female *Pink1−*/− rats were PD and mitophagy and in male *Pink1−*/− rats were PD, pathways of neurodegeneration, and proteasome.

Individual rat transcript copy for each significant gene was correlated to USV acoustic variables. There was one significant negative correlation between Top 10 peak frequency of all calls (Hz) and *Tuba1c* transcript copy (*r* = −0.63, *p* = 0.039, *n* = 11; Figure 10E).

#### Thyroarytenoid transcript expression

Within TA samples, *Pink1* was the most significantly down-regulated gene in *Pink1−*/− rats compared to WT (Table 5 and Figure 9B). There was a smaller list of differentially expressed genes compared to brainstem (*n* = 5). Male *Pink1−*/− rats had more differentially expressed genes (11) compared to *Pink1−*/− females (4); none of which overlapped. Due to small gene lists, enrichment and repurposing analysis was not performed.

**TABLE 5:** TA gene expression differences by genotype.

Gene	Accession #	Direction	*Pink1*−/− vs. WT FDR	*p*-value of: *Pink1*−/− vs. WT	t-statistic of: *Pink1*−/− vs. WT
Pink1	NM_001106694.1	Down	−50.9	0.00000005	−21.86439133
Siglec5	NM_001106249.2	Up	1.56	0.01567048	3.13624167
Spock1	NM_001271297.1	Down	−1.83	0.02894286	−2.64415884
Tg	NM_030988.2	Down	−3.66	0.0302304	−2.52910256
Cers3	NM_001127561.1	Up	1.66	0.04880251	2.59058118

## Discussion

PD is a progressive, neurological disorder that leads to motor and non-motor deficits which significantly impact quality of life; yet the prodromal aspects of the disease are considerably understudied. Genetic rodent models of PD, including the *Pink1−*/− rat, are advantageous to study biological questions that are impossible to address in humans, such as early stage behavioral and gene expression differences between sexes during prodromal disease manifestation. While previous work has evaluated the *Pink1−/−* rat as longitudinal studies and at later ages (i.e., 8 months of age), this study is the first to directly compare young adult (2 months of age) *Pink1−/−* behavior with gene expression changes between sexes and WT controls within the same cohort. The novelty of this study is using a customized NanoString CodeSet specifically created from previously identified gene candidates at 8 months of age (Kelm-Nelson and Gammie, 2020; Lechner et al., 2021). Gene transcripts were probed with the intent to identify whether the same genes are dysregulated at younger ages, prior to the onset of significant classical motor and non-motor signs. We hypothesized that genes involved in degenerative disease and inflammatory pathways would be differentially expressed at 2 months of age and sex-specific differences would be present, regardless of genotype. Due to significant sex- and genotype-differences in body weights, all statistical analyses were designed to covary for weight, but did not alter statistical outcomes. This study reports differences in limb sensorimotor function, cranial motor ultrasonic vocalization behavior, and anxiety as well as gene expression transcript levels in the brainstem and vocal fold (TA) muscle between male and female *Pink1−/−* rats at 2 months of age. These data were used to identify potential gene predictors of prodromal PD pathology, discussed below.

### Female rats demonstrate less anxiety-like behavior in the open field compared to males

Multiple lines of work that suggest individuals diagnosed with PD have increased rates of anxiety and depression, and prevalence is higher in women (Shiba et al., 2000; Quelhas and Costa, 2009; Leentjens et al., 2011; Yamanishi et al., 2013; Altemus et al., 2014; Broen et al., 2016; Cui et al., 2017). Previous rodent studies suggest that male and female *Pink1−/−* rats demonstrate an increase in anxiety-like behavior compared to WT controls (Marquis et al., 2019; Hoffmeister et al., 2021b). The present study also demonstrates genotype and sex differences in anxiety-like behavior in the open field test. All *Pink1−/−* rats entered the center zone significantly fewer times compared to WT rats, but female rats entered the center zone significantly more times compared to male rats. In addition, this study found an interaction effect of genotype and sex on the time spent in the center zone of the open field. While all female rats entered the center zone significantly more times than male rats, only the *Pink1−/−* female rats spent significantly more time in the center zone. Taken together, this data suggests that *Pink1−/−* rats have increased anxiety-like behavior and female rats have decreased anxiety-like behavior at 2 months of age. Additionally, female *Pink1−/−* rats demonstrate significantly less anxiety-like behavior compared to male *Pink1−/−* rats. All *Pink1−/−* rats also displayed a greater total distance traveled compared to WT rats.

While the data from this study are consistent with previous studies that have reported that female rats ambulate more and appear to be less anxious in the open field compared to males (Beatty and Fessler, 1976; Masur et al., 1980), it is important to note that these findings may be driven by the data showing that female *Pink1−/−* rats spent significantly more time in the center zone than all other rats. In addition, because the female rats were only tested in the estrus stage of the estrous cycle, there is a possibility this affected their behavior by enhancing exploratory behavior compared to males. The methods did control for background strain, light exposure, and novelty (only tested once); as these variables have been shown to influence findings in the open field (Miller et al., 2021). Previous *Pink1−*/− rat studies used light-dark box and/or elevated plus maze to track anxiety-like behaviors over time. The present study, while only using one measure, also demonstrates genotype and sex differences in anxiety-like behavior in the open field test. The use of one anxiety test may limit the interpretation of the behavior at this age and future work may want to consider additional behavioral tests.

### Female rats demonstrate increased locomotor activity in the cylinder compared to males

Past data has shown that *Pink1−/−* rats demonstrate sex-specific differences in limb motor function. For example, male *Pink1−/−* rats exhibit limb motor deficits at 8 months of age (e.g., reduced locomotor time across a tapered balance beam, and forelimb and hindlimb movements within the cylinder), while female *Pink1−/−* rats did not exhibit similar limb motor deficits up to 8 months of age (Marquis et al., 2019). The data from this study shows that there are sex differences in limb motor movement at 2 months of age; female rats move around in the cylinder significantly more than male rats. This is consistent with open field locomotor findings. All *Pink1−/−* rats, regardless of sex, demonstrated more spontaneous activity and performed a higher number of forelimb movements in the cylinder.

### *Pink1−/−* rats demonstrate prodromal ultrasonic vocalization dysfunction at 2 months of age

Grant et al. (2015) was the first to quantify the male *Pink1−/−* rat’s development of ultrasonic vocalization dysfunction. Male *Pink1−/−* rats begin to show reductions in bandwidth and peak frequency at 4 and 6 months of age and reduced intensity (loudness) across all tested timepoints. In contrast, Marquis et al. (2019), reported reduced intensity findings in female *Pink1−/−* rats, but no other vocalization deficits by 8 months of age were observable. Here, we report several sex differences and genotype differences in USVs at 2 months of age; this is the first-time vocalizations have been quantified in both sexes and genotypes in the same testing cohorts, which is an important methodological consideration.

Overall, male rats produced significantly more calls than female rats, and *Pink1−/−* rats produced more complex calls than WT rats. The only interaction effect between genotype and sex was for ultrasonic vocalization duration (length of the FM call). WT rats produced significantly longer max duration of FM calls than male *Pink1−/−* rats, but there was no difference in max duration of FM calls in female WT and *Pink1−/−* rats (reviewed in Figure 5B). At 2 months of age, all *Pink1−/−* rats produce FM calls with shorter duration, bandwidth, and peak frequency than WT rats, regardless of sex. In contrast with previous work, this study reports that *Pink1−/−* rats produced FM calls with greater intensity (loudness) than WT rats at 2 months of age. It is important to note that the Grant et al. (2015) and Marquis et al. (2019) papers reported main effects of genotype, collapsed over testing age and not age-specific differences. Sex differences were present for intensity of FM calls with female rats producing FM calls with significantly reduced intensity (loudness) than male rats. In addition, female rats produced FM calls with greater peak frequency than WT rats.

### NanoString gene expression data identifies prodromal differences in transcript levels

Recent work has shown that the global loss of *Pink1* influences gene pathways and neurochemistry within the brainstem periaqueductal gray (PAG) and nucleus ambiguous (AMB), vocal motor brainstem nuclei that control emotional state of vocalizations and vocal fold adduction, respectively (Kelm-Nelson and Gammie, 2020; Lechner et al., 2021). Fostered by these findings in adult rats, the present study evaluated gene expression changes in both the brainstem and TA muscle between male and female *Pink1−/−* rats and control rats at 2 months of age using NanoString technology. The ultimate goal was to discover whether there was tangible overlap between our previous data at 8 months that may constitute early stage markers of prodromal PD in this rodent model. Supplementary Data provided includes the NanoString Code set list, enrichment, and raw data values.

#### Brainstem *Tuba1c* transcript level was identified as a key marker correlated to ultrasonic vocalization at 2 months of age

Out of the 192 genes in our CodeSet, only 41 of those were up- or down-regulated in the whole brainstem. One significantly up-regulated gene of interest is Tubulin Alpha 1c (*Tuba1c*); up-regulation of the protein in *Pink1−*/− females (confirmed with western blot) was also noted in this study. *Tuba1c* is a protein coding gene that is subcellular in the microtubule, binds GTP, and is involved in the Parkin-ubiquitin proteasomal degradation pathway. The loss of Pink1, a key microtubule-interacting protein, causes a disruption of the Pink1/Parkin translocation to mitochondria and is implicated in mitochondrial trafficking, mitochondria turnover, and accumulation of abnormal mitochondria as well as increases in oxidative stress (Gautier et al., 2008). Tuba1c is hypothesized to be a Parkin-dependent ubiquitylation target and interactor of Pink1 (Rakovic et al., 2011; Zanon et al., 2013). Recent evidence has identified the dysregulation of *Tuba1c* as a potential biomarker of PD in a rotenone-induced rat model of PD (Yadav et al., 2022).

Concurrent with this study, overexpression of *Tuba1c* has been found in the AMB (unpublished data), PAG (Kelm-Nelson and Gammie, 2020) and TA muscle (Lechner et al., 2021) of *Pink1−/−* male rats as well as in human motor brainstem regions [male and female datasets (GSE19587)] (Gautier et al., 2008; Lewandowski et al., 2010; Diedrich et al., 2011). Interestingly, observed enrichment in the following biological pathways: Alzheimer’s disease, PD, pathways of neurodegeneration, gap junction, prion disease, and Huntington’s disease, all had *Tuba1c* as a significant up-regulated gene in the enriched pathways.

*Tuba1c* mRNA transcript numbers in the whole brainstem is associated with reduced peak frequency (Hz, average of the top 10 of all calls); *Pink1−*/− rats had generally lower peak frequency measures compared to WT. Peak frequency is suggested to be an important component of short-range communication in the rat where high peak frequencies are related to positive state 50-kHz calls for establishing social proximity and mating behaviors (Brudzynski and Fletcher, 2010; Brudzynski, 2013; Willadsen et al., 2014). Therefore, identifying this gene points to a functional mechanism of action in the CNS leading to prodromal behavioral differences in vocal communication.

#### Fewer differences in transcript levels exist in the thyroarytenoid muscle of all *Pink1−/−* rats

In the TA muscle, for all *Pink1−/−* rats, there were few dysregulated genes. There were three down-regulated genes (*Pink1*, *Spock1*, *Tg*) and two up-regulated genes (*Siglec5*, *Cers3*). Female *Pink1−/−* had no up-regulated genes and 4 down-regulated genes, while male *Pink1−/−* rats had 8 up-regulated genes and 3 down-regulated genes. This study shows that as early as 2 months of age, male *Pink1−/−* rats already display an up-regulation of nuclear factor kappa beta subunit 1 (*Nfkb1*), which was also up-regulated in the TA muscle at 8 months of age (Lechner et al., 2021). In addition, steroid 5 alpha-reductase 1 (*Srd5a1*), which plays a significant role in androgen/testosterone metabolism, is up-regulated in female *Pink1−/−* rats. Due to the limited number of genes in the NanoString CodeSet, it is possible that there were more differences in mRNA transcript levels in the TA muscle at 2 months of age than identified in this study. Additionally, the sample size for NanoString in this study was only 4 per group, which may have been too small to detect other differences in transcript levels.

## Conclusion

In summary, this work examined the male-female differences in the behavior and gene expression changes at 2 months of age in the *Pink1−/−* rat model within the same cohort. These data are consistent with previous studies that demonstrate differences in ultrasonic vocalization performance and anxiety-like behavior precede the development of any limb motor deficits in the *Pink1-/-* model, which is analogous to idiopathic PD in humans. These metrics provide the basis for studying prodromal behavior in this model. However, to fully characterize the prodrome in this model and more accurately develop early predictors of PD pathology, future studies should include behavioral and biochemical analyses of olfactory and sleep patterns. Gene expression data from this study demonstrates a significant difference in the number of genes and types of genes that are dysregulated in male and female *Pink1−/−* rats. Several genes of interest, including *Tuba1c*, were identified as potential targets for future drug repurposing and vocal therapy studies.

## Data availability statement

The original contributions presented in this study are included in the article/Supplementary material, further inquiries can be directed to the corresponding author/s.

## Ethics statement

The animal study was reviewed and approved by the University of Wisconsin-Madison Institutional Animal Care and Use Committee (IACUC).

## Author contributions

CK-N conceived and designed the study, extracted RNA, performed NanoString preparation, and analysis. CK-N, SL, JW, NP, TK, and AR performed animal work and harvested tissues. SL, JW, NP, TK, and AR analyzed data files. CK-N and SL performed statistical analyses. CK-N, SL, and JW interpreted results and wrote the manuscript. All authors approved of final manuscript.
