# Supplementary material for: Predictors of prodromal Parkinson’s disease in young adult Pink1−/− rats
Source: Front Behav Neurosci. 2022 Sep 12;16:867958. doi: 10.3389/fnbeh.2022.867958 (PMC9510667; doi:10.3389/fnbeh.2022.867958)
Supplement: Supplementary file 1 [file Table_1.DOCX]

**Supplementary Table 1**: *All calls – Means (SEM).*

|  | **Acoustic parameter/unit** | **Male** | | **Female** | |
| --- | --- | --- | --- | --- | --- |
|  |  | WT | *Pink1-/-* | WT | *Pink1-/-* |
| Average | Duration (sec) | 0.040 (0.002) | 0.034 (0.003) | 0.035 (0.005) | 0.030 (0.002) |
|  | Bandwidth (Hz) | 17339.09 (1184.97) | 12770.13 (938.88) | 13976.32 (1058.48) | 13494.21 (1391.35) |
|  | Intensity (dB) | -48.69 (0.67) | -48.45 (0.55) | -52.20 (1.01) | -51.25  (0.72) |
|  | Peak Frequency (Hz) | 53658.58 (806.33) | 48473.66 (1746.11) | 61463.31 (1354.51) | 56563.62  (583.66) |
| Maximum | Duration | 0.209  (0.026) | 0.254 (0.040) | 0.092 (0.025) | 0.140 (0.026) |
|  | Bandwidth | 55341.67 (2959.56) | 37960.00 (3123.47) | 43120.00 (3139.07) | 36520.00  (3072.27) |
|  | Intensity | -31.61 (0.87) | -30.55 (1.10) | -33.96 (1.55) | -35.20 (1.21) |
|  | Peak Frequency | 71608.33 (1701.40) | 67320.00 (1810.70) | 78480.00 (1508.77) | 73850.00 (1422.15) |
| Top 10 | Duration | 0.112 (0.014) | 0.113 (0.016) | 0.066 (0.014) | 0.078 (0.016) |
|  | Bandwidth | 41339.17 (2789.90) | 29602.00 (1982.94) | 31542.00 (1724.99) | 29217.00 (2573.54) |
|  | Intensity | -35.26 (0.90) | -34.87 (0.57) | -39.99 (1.35) | -39.34 (1.27) |
|  | Peak Frequency | 66542.50 (1362.47) | 62272.00 (2437.87) | 73275.00 (1406.18) | 68581.00 (1529.99) |

**Supplementary Table 1**: Mean (standard error of the mean(SEM)) for acoustic parameters of all ultrasonic vocalizations for each genotype and sex. Abbreviations: sec=second, Hz=Hertz, dB=decibel.
