# Supplementary material for: Predictors of prodromal Parkinson’s disease in young adult Pink1−/− rats
Source: Front Behav Neurosci. 2022 Sep 12;16:867958. doi: 10.3389/fnbeh.2022.867958 (PMC9510667; doi:10.3389/fnbeh.2022.867958)
Supplement: Supplementary file 2 [file Table_2.DOCX]

**Supplementary Table 2**: *All calls – interaction effects f & p values.*

|  | **Acoustic parameter/unit** | **Genotype x Sex** |
| --- | --- | --- |
| Average | Duration (sec) | F(1, 38) = 1.092, p = 0.303 |
|  | Bandwidth (Hz) | F(1, 38) = 3.068, p = 0.088 |
|  | Intensity (dB) | F(1, 38) = 0.226, p = 0.637 |
|  | Peak Frequency (Hz) | F(1, 38) = 0.048, p = 0.828 |
| Maximum | Duration | F(1, 38) = 0.002, p = 0.961 |
|  | Bandwidth | F(1, 38) = 3.056, p = 0.089 |
|  | Intensity | F(1, 38) = 0.937, p = 0.339 |
|  | Peak Frequency | F(1, 38) = 0.011, p = 0.917 |
| Top 10 | Duration | F(1, 38) = 0.205, p = 0.653 |
|  | Bandwidth | F(1, 38) = 3.911, p = 0.055 |
|  | Intensity | F(1, 38) = 0.015, p = 0.903 |
|  | Peak Frequency | F(1, 38) = 0.015, p = 0.902 |

**Supplementary Table 2**: Interaction effect f and *p-*values for acoustic parameters of all ultrasonic vocalizations. Abbreviations: sec=second, Hz=Hertz, dB=decibel.
