# Supplementary material for: Predictors of prodromal Parkinson’s disease in young adult Pink1−/− rats
Source: Front Behav Neurosci. 2022 Sep 12;16:867958. doi: 10.3389/fnbeh.2022.867958 (PMC9510667; doi:10.3389/fnbeh.2022.867958)
Supplement: Supplementary file 3 [file Table_3.DOCX]

**Supplementary Table 3**: *All calls – main effects f & p values.*

|  | **Acoustic parameter/unit** | **Genotype** | **Sex** |
| --- | --- | --- | --- |
| Average | Duration (sec) | F(1, 38) = 3.112, p = 0.086 | F(1, 38) = 5.883, p = 0.020 |
|  | Bandwidth (Hz) | F(1, 38) = 4.686, p = 0.037 | F(1, 38) = 1.279, p = 0.265 |
|  | Intensity (dB) | F(1, 38) = 0.619, p = 0.436 | F(1, 38) = 17.60, p < 0.001 |
|  | Peak Frequency (Hz) | F(1, 38) = 11.46, p = 0.002 | F(1, 38) = 51.56, p < 0.001 |
| Maximum | Duration | F(1, 38) = 2.485, p = 0.123 | F(1, 38) = 15.21, p < 0.001 |
|  | Bandwidth | F(1, 38) = 15.12, p < 0.001 | F(1, 38) = 4.907, p = 0.033 |
|  | Intensity | F(1, 38) = 0.006, p = 0.937 | F(1, 38) = 8.728, p = 0.005 |
|  | Peak Frequency | F(1, 38) = 7.410, p = 0.010 | F(1, 38) = 16.73, p < 0.001 |
| Top 10 | Duration | F(1, 38) = 0.287, p = 0.595 | F(1, 38) = 11.49, p = 0.002 |
|  | Bandwidth | F(1, 38) = 8.731, p = 0.005 | F(1, 38) = 4.578, p = 0.039 |
|  | Intensity | F(1, 38) = 0.239, p = 0.628 | F(1, 38) = 18.98, p < 0.001 |
|  | Peak Frequency | F(1, 38) = 6.817, p = 0.013 | F(1, 38) = 14.43, p < 0.001 |

**Supplementary Table 3**: Interaction effect f and *p-*values for acoustic parameters of simple ultrasonic vocalizations. Abbreviations: sec=second, Hz=Hertz, dB=decibel.
