# Supplementary material for: Predictors of prodromal Parkinson’s disease in young adult Pink1−/− rats
Source: Front Behav Neurosci. 2022 Sep 12;16:867958. doi: 10.3389/fnbeh.2022.867958 (PMC9510667; doi:10.3389/fnbeh.2022.867958)
Supplement: Supplementary file 4 [file Table_4.DOCX]

**Supplementary Table 4**: *Simple calls – Means (SEM).*

|  | **Acoustic parameter/unit** | **Male** | | **Female** | |
| --- | --- | --- | --- | --- | --- |
|  |  | WT | *Pink1-/-* | WT | *Pink1-/-* |
| Average | Duration (sec) | 0.027 (0.002) | 0.024 (0.003) | 0.023 (0.003) | 0.020 (0.002) |
|  | Bandwidth (Hz) | 6372.04 (276.83) | 3749.09 (253.84) | 5883.79 (539.55) | 4332.34 (418.50) |
|  | Intensity (dB) | -50.56 (0.66) | -51.50 (0.57) | -53.20 (1.23) | -53.48 (1.22) |
|  | Peak Frequency (Hz) | 51251.32 (423.69) | 45102.25 (1518.42) | 56424.06 (1187.19) | 53365.47 (1269.78) |
| Maximum | Duration | 0.083  (0.021) | 0.110 (0.022) | 0.050 (0.009) | 0.069 (0.013) |
|  | Bandwidth | 15275.00 (911.64) | 8690.00 (1023.88) | 12410.00 (855.11) | 8570.00 (799.17) |
|  | Intensity | -34.95 (1.07) | -36.69 (1.55) | -38.40 (1.14) | -42.84 (2.05) |
|  | Peak Frequency | 62650.00 (1544.27) | 59440.00 (2369.63) | 66150.00 (1670.15) | 66400.00 (2432.51) |
| Top 10 | Duration | 0.044 (0.004) | 0.038 (0.006) | 0.032 (0.005) | 0.027 (0.004) |
|  | Bandwidth | 10219.17 (615.56) | 5127.00 (489.56) | 8315.00 (662.89) | 5452.54 (780.19) |
|  | Intensity | -41.45 (1.18) | -47.19 (1.04) | -47.09 (1.35) | -50.34 (1.94) |
|  | Peak Frequency | 55521.67 (612.08) | 49682.00 (2480.79) | 59996.00 (1549.24) | 56012.86 (1463.45) |

**Supplementary Table 4**: Mean (standard error of the mean (SEM)) for acoustic parameters of simple ultrasonic vocalizations for each genotype and sex. Abbreviations: sec=second, Hz=Hertz, dB=decibel.
