# Supplementary material for: Predictors of prodromal Parkinson’s disease in young adult Pink1−/− rats
Source: Front Behav Neurosci. 2022 Sep 12;16:867958. doi: 10.3389/fnbeh.2022.867958 (PMC9510667; doi:10.3389/fnbeh.2022.867958)
Supplement: Supplementary file 5 [file Table_5.DOCX]

**Supplementary Table 5**: *Simple calls – interaction effects f & p values.*

|  | **Acoustic parameter/unit** | **Genotype x Sex** |
| --- | --- | --- |
| Average | Duration (sec) | F(1, 38) = 0.485, p = 0.490 |
|  | Bandwidth (Hz) | F(1, 38) = 4.454, p = 0.041 |
|  | Intensity (dB) | F(1, 38) = 0.119, p = 0.732 |
|  | Peak Frequency (Hz) | F(1, 38) = 1.882, p = 0.178 |
| Maximum | Duration | F(1, 38) = 0.060, p = 0.809 |
|  | Bandwidth | F(1, 38) = 2.282, p = 0.139 |
|  | Intensity | F(1, 38) = 0.834, p = 0.367 |
|  | Peak Frequency | F(1, 38) = 0.740, p = 0.395 |
| Top 10 | Duration | F(1, 38) = 0.838, p = 0.366 |
|  | Bandwidth | F(1, 38) = 2.970, p = 0.093 |
|  | Intensity | F(1, 38) = 0.786, p = 0.381 |
|  | Peak Frequency | F(1, 38) = 0.097, p = 0.757 |

**Supplementary Table 5**: Interaction effect f and *p-*values for acoustic parameters of simple ultrasonic vocalizations. Abbreviations: sec=second, Hz=Hertz, dB=decibel.
