# Supplementary material for: Predictors of prodromal Parkinson’s disease in young adult Pink1−/− rats
Source: Front Behav Neurosci. 2022 Sep 12;16:867958. doi: 10.3389/fnbeh.2022.867958 (PMC9510667; doi:10.3389/fnbeh.2022.867958)
Supplement: Supplementary file 6 [file Table_6.DOCX]

**Supplementary Table 6**: *Simple calls – main effects f & p values.*

|  | **Acoustic parameter/unit** | **Genotype** | **Sex** |
| --- | --- | --- | --- |
| Average | Duration (sec) | F(1, 38) = 1.042, p = 0.314 | F(1, 38) = 3.358, p = 0.075 |
|  | Bandwidth (Hz) | F(1, 38) = 43.74, p < 0.001 | F(1, 38) = 0.011, p = 0.919 |
|  | Intensity (dB) | F(1, 38) = 0.414, p = 0.524 | F(1, 38) = 5.928, p = 0.020 |
|  | Peak Frequency (Hz) | F(1, 38) = 16.70, p < 0.001 | F(1, 38) = 35.57, p < 0.001 |
| Maximum | Duration | F(1, 38) = 4.126, p = 0.049 | F(1, 38) = 3.643, p = 0.064 |
|  | Bandwidth | F(1, 38) = 32.91, p < 0.001 | F(1, 38) = 2.698, p = 0.109 |
|  | Intensity | F(1, 38) = 4.387, p = 0.043 | F(1, 38) = 10.60, p = 0.002 |
|  | Peak Frequency | F(1, 38) = 0.542, p = 0.466 | F(1, 38) = 6.763, p = 0.013 |
| Top 10 | Duration | F(1, 38) = 1.520, p = 0.225 | F(1, 38) = 8.108, p = 0.007 |
|  | Bandwidth | F(1, 38) = 37.80, p < 0.001 | F(1, 38) = 1.489, p = 0.230 |
|  | Intensity | F(1, 38) = 10.23, p = 0.003 | F(1, 38) = 9.775, p = 0.003 |
|  | Peak Frequency | F(1, 38) = 6.596, p = 0.014 | F(1, 38) = 9.548, p = 0.004 |

**Supplementary Table 6**: Interaction effect f and *p-*values for acoustic parameters of all ultrasonic vocalizations. Abbreviations: sec=second, Hz=Hertz, dB=decibel.
