# Supplementary material for: Predictors of prodromal Parkinson’s disease in young adult Pink1−/− rats
Source: Front Behav Neurosci. 2022 Sep 12;16:867958. doi: 10.3389/fnbeh.2022.867958 (PMC9510667; doi:10.3389/fnbeh.2022.867958)
Supplement: Supplementary file 8 [file Table_8.DOCX]

**Supplementary Table 8**: *Frequency modulated (FM) calls – interaction effects f & p values.*

|  | **Acoustic parameter/unit** | **Genotype x Sex** |
| --- | --- | --- |
| Average | Duration (sec) | F(1, 38) = 2.207, p = 0.146 |
|  | Bandwidth (Hz) | F(1, 38) = 1.117, p = 0.297 |
|  | Intensity (dB) | F(1, 38) = 0.201, p = 0.657 |
|  | Peak Frequency (Hz) | F(1, 38) = 0.016, p = 0.900 |
| Maximum | Duration | F(1, 38) = 4.908, p = 0.033 |
|  | Bandwidth | F(1, 38) = 1.524, p = 0.225 |
|  | Intensity | F(1, 38) = 0.980, p = 0.328 |
|  | Peak Frequency | F(1, 38) = 0.962, p = 0.333 |
| Top 10 | Duration | F(1, 38) = 2.060, p = 0.159 |
|  | Bandwidth | F(1, 38) = 2.373, p = 0.132 |
|  | Intensity | F(1, 38) = 0.119, p = 0.732 |
|  | Peak Frequency | F(1, 38) = 2.435, p = 0.127 |

**Supplementary Table 8**: Interaction effect f and *p-*values for acoustic parameters of FM ultrasonic vocalizations. Abbreviations: sec=second, Hz=hertz, dB=decibel.
