# Supplementary material for: Predictors of prodromal Parkinson’s disease in young adult Pink1−/− rats
Source: Front Behav Neurosci. 2022 Sep 12;16:867958. doi: 10.3389/fnbeh.2022.867958 (PMC9510667; doi:10.3389/fnbeh.2022.867958)
Supplement: Supplementary file 9 [file Table_9.DOCX]

**Supplementary Table 9**: *Frequency modulated (FM) calls – main effects f & p values.*

|  | **Acoustic parameter/unit** | **Genotype** | **Sex** |
| --- | --- | --- | --- |
| Average | Duration (sec) | F(1, 38) = 15.80, p < 0.001 | F(1, 38) = 0.340, p = 0.563 |
|  | Bandwidth (Hz) | F(1, 38) = 13.03, p < 0.001 | F(1, 38) = 0.767, p = 0.387 |
|  | Intensity (dB) | F(1, 38) = 10.50, p = 0.002 | F(1, 38) = 8.760, p = 0.005 |
|  | Peak Frequency (Hz) | F(1, 38) = 47.96, p < 0.001 | F(1, 38) = 51.65, p < 0.001 |
| Maximum | Duration | F(1, 38) = 1.687, p = 0.202 | F(1, 38) = 0.512, p = 0.479 |
|  | Bandwidth | F(1, 38) = 8.917, p = 0.005 | F(1, 38) = 3.385, p = 0.074 |
|  | Intensity | F(1, 38) = 1.213, p = 0.278 | F(1, 38) = 5.915, p = 0.020 |
|  | Peak Frequency | F(1, 38) = 22.13, p < 0.001 | F(1, 38) = 10.59, p = 0.002 |
| Top 10 | Duration | F(1, 38) = 4.967, p = 0.032 | F(1, 38) = 1.674, p = 0.204 |
|  | Bandwidth | F(1, 38) = 7.279, p = 0.010 | F(1, 38) = 1.929, p = 0.173 |
|  | Intensity | F(1, 38) = 2.055, p = 0.160 | F(1, 38) = 14.52, p < 0.001 |
|  | Peak Frequency | F(1, 38) = 36.70, p < 0.001 | F(1, 38) = 16.50, p < 0.001 |

**Supplementary Table 9**: Interaction effect f and *p-*values for acoustic parameters of FM ultrasonic vocalizations. Abbreviations: sec=second, Hz=Hertz, dB=decibel.
